# What Role Might Non-Mating Receptors Play in *Schizophyllum commune*?

**DOI:** 10.3390/jof7050399

**Published:** 2021-05-20

**Authors:** Sophia Wirth, Daniela Freihorst, Katrin Krause, Erika Kothe

**Affiliations:** Friedrich Schiller University Jena, Institute of Microbiology, Microbial Communication, 25 07743 Neugasse Jena, Germany; sophia.wirth@uni-jena.de (S.W.); danielafreihorst@aol.com (D.F.); erika.kothe@uni-jena.de (E.K.)

**Keywords:** *Schizophyllum commune*, pheromone receptor-like genes, *B* mating-type locus, mating, self-recognition

## Abstract

The *B* mating-type locus of the tetrapolar basidiomycete *Schizophyllum commune* encodes pheromones and pheromone receptors in multiple allelic specificities. This work adds substantial new evidence into the organization of the *B* mating-type loci of distantly related *S. commune* strains showing a high level of synteny in gene order and neighboring genes. Four pheromone receptor-like genes were found in the genome of *S. commune* with *brl1, brl2* and *brl3* located at the *B* mating-type locus, whereas *brl4* is located separately. Expression analysis of *brl* genes in different developmental stages indicates a function in filamentous growth and mating. Based on the extensive sequence analysis and functional characterization of *brl*-overexpression mutants, a function of Brl1 in mating is proposed, while Brl3, Brl4 and Brl2 (to a lower extent) have a role in vegetative growth, possible determination of growth direction. The *brl3* and *brl4* overexpression mutants had a dikaryon-like, irregular and feathery phenotype, and they avoided the formation of same-clone colonies on solid medium, which points towards enhanced detection of self-signals. These data are supported by localization of Brl fusion proteins in tips, at septa and in not-yet-fused clamps of a dikaryon, confirming their importance for growth and development in *S. commune*.

## 1. Introduction

*Schizophyllum commune* is a well-known wood-decaying basidiomycete [1]. Unlike many other basidiomycetes, *S. commune* can be cultured in the laboratory on artificial media and can be genetically modified, allowing the intensive studying of mating and mushroom development at the molecular level [2,3].

The tetrapolar mating system of *S. commune* is not required for the initial fusion between mating partners but is involved in the stable formation of a dikaryotic mycelium involving nuclear migration and clamp cell fusion [4]. With two unlinked multigenic mating-type loci, the tetrapolar mating system of *S. commune* is conserved in basidiomycetes including *Coprinopsis cinerea* [5], *Laccaria bicolor* [6], *Flammulina velutipes* [7] and *Ustilago maydis* [8]. The *A* locus encodes a set of divergently transcribed homeodomain transcription factors [9], while the *B* locus comprises pheromone receptors surrounded by several pheromone precursor genes [3,10]. Generally, two allelic versions of pheromone receptors occur in each *S. commune* strain, which thereby define the specificity of the locus by recognizing either Bα or Bβ pheromones.

The publicly available *S. commune* genome sequences allow a comparison of different *S. commune* strains from different geographic locations and with unknown mating-type loci organization and specificities. All annotated basidiomycete pheromone receptors are orthologs of Ste3 of *Saccharomyces cerevisiae*, which is also true for the Bα and Bβ mating receptors of *S. commune* (Bar and Bbr, respectively) [11]. Generally, these proteins are characterized by the presence of seven transmembrane domains and their association with intracellular G-proteins (GPCR) triggering signaling pathways to coordinate *B*-dependent development. They are further characterized by a short N-terminal extracellular domain and a long cytoplasmic C-terminal tail. Binding of a pheromone from a non-self mating partner stimulates the associated heterotrimeric G-proteins to dissociate into the Gα and Gβγ subunits, which trigger the subsequent G-protein signaling pathways. The specificities, and hence sequences, of the strains differ to allow for different pheromones to be recognized [12,13]. A strain carrying a deletion of the *B* receptor and pheromone genes that shows no *B*-regulated development in matings with any wild-type strain is available. Monokaryotic mutant strains with constitutively active *B* mating-type genes exist as well, which show an aberrant phenotype typically found in semi-compatible mating [10].

Among the 75 encoded proteins with predicted transmembrane domains [14], six fungal pheromone receptors are found in a given strain. Since mating-type specificities are different, allelic versions of these six pheromone receptors are to be considered. In any genome, only the Bar and Bbr mating receptors have been functionally characterized. Of these, specifically the pheromone receptors Bbr2 [10] and Bar2 [11], as well as Bbr1 [15], have received the most attention. These true pheromone-recognizing receptors induce pheromone/receptor-dependent development resulting in fertile dikaryons. The remaining four *B*-receptor-like genes per genome (*brl1* through *brl4*) are orthologs of *S. cerevisiae* Ste3 a-factor receptor, with three (*brl1–3*) surrounding the *bar* and *bbr* gene loci within an 81 kb region, while *brl4* was identified on a separate scaffold [11]. The *brl* genes are not surrounded by pheromone genes, indicating that they are not directly associated with mating. In addition, they are highly similar in sequence, which indicates that they do not differ regarding recognition of ligands. This finding is in line with the identification of pheromone receptor-like genes in the genome of many basidiomycetes [16,17,18], including *F. velutipes* [7], *Postia placenta* [19] and *Phanerochaete chrysosporium* [20], and the mycorrhizal species *L. bicolor* [6]. To unravel the function of the *B* receptor-like genes, expression analysis in *L. bicolor* showed association with different life stages [6]. For the receptor-like Crp2 protein from *C. neoformans*, a role in cell fusion and sporulation has been postulated, since it activates the same G-protein-coupled signaling pathway as do the true mating-type receptors [21].

Here, it could be established that overexpression of the pheromone receptor-like genes *brl3 and brl4* increase self-sensing of *S. commune* and induce an asymmetrical colony growth, while the function of *brl1* could be attributed to an early mating response.

## 2. Materials and Methods

### 2.1. Culture Conditions and Strains

*S. commune* strains (Table S1) were routinely grown on minimal medium (MM [22]) with the addition of 4 mM tryptophan for *trp^-^* strains and 0.1 mM uracil for *ura^-^* strains at 28 °C. The sister strains *S. commune* 4–39 and W22 are derived from a lineage of 40 back-crosses and are co-isogenic, differing only in mating-type to allow for out-crossing to *S. commune* 12–43. To assess growth and biomass formation on other carbon sources, glucose was replaced by 4% xylose or 3.4% sucrose, and colonies were grown on agar medium covered with a cellophane foil for easy biomass recovery using five biological replicates. Mycelium was dried overnight and weighed. Statistical analysis was done with a paired, two-tailed t-test. A modified sandwich system was used to grow compatible monokaryons for mating interactions allowing mycelial harvesting 6, 12, 24 and 48 h after mating and in the 8-day-old, established dikaryon [23].

For growth in liquid culture, a mycelial homogenate was prepared using a laboratory blender (neoLab, Heidelberg, Germany), and cultures were grown at 250 rpm in 500 mL Erlenmeyer flasks containing 200 mL MM.

### 2.2. Gene Overexpression and Protein Labeling

Overexpression vectors for *brl1* (ID: 2638177)*, brl2* (ID: 2704867)*, brl3* (ID: 2638261) and *brl4* (ID: 2691538) were constructed using yeast recombinational cloning with the binary shuttle vector pRS415 carrying the *leu2* gene for yeast selection and the *bla* gene for *E. coli* selection [24]. For overexpression, *brl* genes and a 758 bp long fragment of the *tef1* promoter (elongation factor 1α; ID: 84142) were amplified from genomic DNA of *S. commune* H4–8 by PCR using PhusionTaq (Thermo Fisher Scientific, Waltham, MA, USA) and specific primer pairs (Table S2). PCR products were recombined in the vector pRS415 (linearized with *Hind*III and *Bam*HI) by using overhangs of at least 30 bp. The plasmids were isolated from the transformed yeast cells [25] and checked by PCR (M13 standard primers), and the correct fusion product was verified by sequencing (GATC Biotech, Konstanz, Germany). Electrocompetent *E. coli* were transformed using 1 µL of DNA, followed by colony PCR verification and plasmid isolation (GeneJET Plasmid-Miniprep-Kit, Thermo Fisher Scientific, Waltham, USA). Restriction with *Bam*HI, *Sal*I and *Hind*III was used to confirm the expected products, pRSbrl1OE, pRSbrl2OE, pRSbrl3OE and pRSbrl4OE. Subsequently, plasmid DNA was transformed into protoplasts of the tryptophan and uracil auxotrophic strain *S. commune* T33 [26], and successful transformation was verified by sequencing (GATC Biotech, Konstanz, Germany). An empty vector control (evc) was used for control.

For protein labeling, *brl1* and *brl2* were amplified from genomic DNA of strain H4–8 including ca. 500 bp of the native promoter. The promoter regions of the genes were analyzed by MEME server before primer design. The reverse primer for each receptor gene amplification included the sequences for a myc tag and His tag. The PCR amplicons (for primers, see Table S2) were generated by proofreading polymerase Q5 Hot Start (NEB) and cloned into pJET1.2/blunt (Thermo Fisher Scientific, Waltham, MA, USA). Plasmids were sequenced to verify the correct sequence and tag presence at the 3` end. The *S. commune* selection marker gene *ble* was introduced into *Xba*I sites of pJET1.2/blunt vector, resulting in the vectors pbrl1myc and pbrl2His. Subsequently, constructs were introduced into *S. commune* T33 and transformants selected for 4 days at 30 °C on MM plates containing 4 mM tryptophan, 10 mM uracil and 15 μg mL^−1^ phleomycin. Successful transformation was verified by sequencing (GATC Biotech, Konstanz, Germany).

### 2.3. In Silico Analyses

The genome sequence of *S. commune* strains H4–8, TatD and LoeD was screened for the presence of pheromone receptor genes by blast searches, and gene models for pheromone receptor genes were manually annotated. The promoter sequences of the *brl* genes and *bar3* and *bbr2* (approximately 1000 bp upstream of ATG) were investigated by Multiple Em for Motif Elicitation (MEME) [27] and Motif Alignment & Search Tool (MAST) [28]. The predicted motifs were analyzed in detail using Tomtom Motif Comparison Tool v4.10.1 [28].

Secondary protein structures of Brl1, Brl2, Brl3 and Brl4 encoded proteins were predicted using the online server I-TASSER and GPCR-I-TASSER [29,30,31,32]. Proteins were submitted without putative signal peptides which were cut from sequence after prediction by SignalP server [33].

Transmembrane domains were predicted using Split4.0 [34], Kyte and Doolittle algorithm [35], the MPEx software (http://blanco.biomol.uci.edu/mpex: accessed on 28 March 2021) based upon Kyte and Doolittle (1982) and others [36] and the TMHMM server tool [37].

The software ChromoMapper [38] was used to display the *bar* and *bbr* loci compared to the three available genome sequences. Conceptually translated proteins were used for Blast searches to display similarities. The DNA and protein sequences were analyzed with MAFFT alignments using the G-INS-i strategy and BLOSUM80 scoring matrix especially for proteins [39,40], and similarity was calculated using BioEdit.

### 2.4. Quantitative PCR

Total RNA was isolated from *S. commune* using the RNeasy Plant Mini Kit (Qiagen, Hilden, Germany) according to the manufacturer’s instructions, and RNA concentration was measured spectrophotometrically (NanoVue Photometer, GE Healthcare, Chicago, IL USA). For reverse transcription, the QuantiTect Reverse Transcription Kit (Qiagen, Hilden, Germany) was used with Maxima SYBR Green 2x Master Mix (Thermo Fisher Scientific, Waltham, USA) in a total volume of 12.5 µL. Each reaction mix contained gene-specific primer pairs (0.5 µL each of 10 pmol/µL stocks; see Table S2), 2 µL cDNA, 3.25 µL *A. dest.* and 6.25 µL SYBR Green 2x Master Mix. For each primer pair, the amplification efficiency was calculated using a cDNA dilution. Measurements took place in 48-well white PCR plates (Multiplate Low-Profile Unskirted PCR Plates, BioRad, Hercules, USA) sealed with optical foil (Microseal ‘C’ Optical Seals, BioRad, Hercules, CA, USA). The reaction was performed in a MiniOpticon cycler (BioRad, Hercules, CA, USA) with initial denaturation at 95 °C for 10 min and 35 cycles (95 °C for 15 s, 55–60 °C for 10–23 s, 72 °C for 30 s) followed by melting curve analysis. Three biological and three technical replicates and two controls, one without reverse transcriptase and one without template DNA, were used in every run. The genes *act1, tef1* and *ubi* were used as reference genes. The ct values of target genes were normalized with respect to the reference genes and calculated for relative and normalized fold change by the equation 2-ΔΔCt [41,42].

### 2.5. Whole-Genome Microarrays

The microarrays were carried out as previously described [11] using 50-mer oligos for all 13,181 predicted genes of S. commune H4–8. Whole RNA was labelled with biotin, and 1 μg of total RNA was used for each array. The preprocessing of the data was done using LIMMA packages of the Bioconductor software [43]. For background correction, the intensities of blank probes were used (single T nucleotides). The resulting median background intensity was then subtracted from the actual spot intensity values. Any negative value was converted into a low positive value, and signal intensities were log2 transformed. Replicates were averaged, and the data were processed by quantile normalization. Statistical and quality tests were done. A p value in Student’s t-test under 0.05 was set to show significant differential expression values. Microarray data were deposited in NCBI’s Gene Expression Omnibus webpage and are accessible through Platform GPL11376 and GEO Series accession number GSE26401 (http://www.ncbi.nlm.nih.gov/geo/query/acc.cgi?accGSE26401; accessed on 28 March 2021).

### 2.6. Phylogenetic Analysis

Fungal pheromone receptor protein and DNA sequences were selected using the NCBI database, Unite or the respective genome databases (Joint Genome Institute, Walnut Creek, USA). Alignments were made with the server MAFFT version 7 [39,40], using the F-INS-i or G-INS-i strategies and BLOSUM80 as well as the unalign-level-setting 0.0 or 0.8. The software MrBayes 3.2.2. [44] and Markov chain Monte Carlo method (Dayhoff modelling) were used for protein tree calculation with the following settings: prset aamodelpr = fixed(dayhoff); prset ratepr = variable; mcmcp nruns = 2 ngen = 3,000,000 printfreq = 1000 samplefreq = 100 nchains = 4; mcmc. The mixed trees were calculated using these settings: prset applyto = (2) ratepr = variable; lset applyto = (2) nst = 6 rates = gamma; prset applyto = (1) aamodelpr = fixed(dayhoff); prset applyto = (all) ratepr = variable; unlink statefreq = (all) revmat = (all) shape = (all) pinvar = (all); mcmcp nruns = 2 ngen = 1,000,000 printfreq = 1000 samplefreq = 100 nchains = 4; mcmc; sumt burnin = 2000.

The Cyberinfrastructure for Phylogenic Research (CIPRES) server was used for computing power (http://www.phylo.org/index.php; accessed last on 28 March 2021). The software FigTree v1.4.2 and Inkscape 0.91 were chosen to edit the resulting trees [45], while Tracer software was used to verify the results [46].

### 2.7. Immunofluorescence Staining

Immunofluorescence staining of epitope-tagged proteins was performed according to [11]. In brief, methanol (HPLC grade) was added and incubated for 10 min at −20 °C. Mycelium was fixed with 3.7% formaldehyde in PME (50 mM PIPES pH 6.7, 25 mM EGTA pH 8.0, 5 mM MgSO_4_) for 90 min at room temperature. Three times washing with PME followed, the last time for 10 min. Then, 30 mg lysing enzyme of *T. harzianum* (Sigma-Aldrich, Taufkirchen, Germany) was added to egg white in PME, mixed vigorously, and incubated for 15 min at 37 °C to solve and activate the enzyme. The lysing solution was added to the mycelium and incubated for 20 to 30 min at room temperature. Three times washing with PBS (137 mM NaCl, 2.7 mM KCl, 8 mM Na_2_HPO_4_, 1.8 mM KH_2_PO_4_), the last time for 5 min, and then a permeabilization with PBS + 0.3% Triton X-100 for 10 min followed. The wash steps were done again with PBS. Unspecific binding was blocked by 1 to 3% BSA in PBS (5 min at 37 °C). The first antibody was 1:200 diluted in PBS + 1 to 3% BSA and incubated on the mycelium overnight at 4 °C. Wash steps were carried out with PBS (last time 10 min), and the second antibody, which was FITC- or TRITC-coupled, was added (1:100 diluted in PBS + 1 to 3% BSA) for 60 min at 37 °C in the dark. Again, wash steps were carried out with PBS, and the coverslips were embedded upside down into freshly made embedding medium (0.1 M Tris/HCl pH 8.0, 50% glycerol, 1 mg/mL phenylene diamine, 0.1 mg/mL DAPI). An incubation of at least 24 h at 4 °C followed, before microscopy ensued.

### 2.8. Microscopy

Hyphae of *S. commune* were routinely grown on coverslips which were laid on top of solid medium for five to ten days at 30 °C. For early dikaryons, two coverslips of the mating-type partners were sandwiched and stained after 24 h (incubation on medium). Morphology of hyphae was studied with the Axioplan 2 microscope (Carl Zeiss, Jena, Germany). Images were taken with the digital camera system Insight Firewire 4 (Diagnostic Instruments, Sterling Heights, MI, USA) and the software Spot Version 4.6 (Diagnostic Instruments). For detailed imaging of hyphae, the laser scanning microscope LSM780 (Carl Zeiss, Jena, Germany) was used with GaAsP-detector and software ZEN black. All immunofluorescence specimens were grown on special coverslips (high performance, D = 0.17 mm ± 0.005 mm, Carl Zeiss, Jena, Germany).

## 3. Results

### 3.1. In Silico Analyses of brl Genes from Different Genotypes

**Figure 1:**
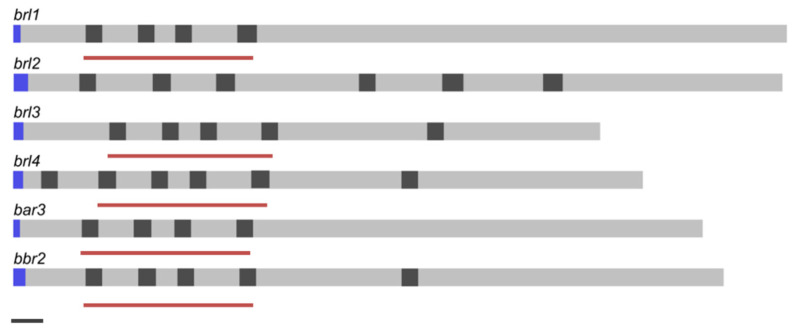
Gene structure of *ste3*-like genes of *S. commune* strain H4–8. Exons (grey), introns (dark grey), signal peptide (blue) and scaling according to JGI annotation. Black bar (bottom left) indicates 100 bp. Red bars indicate similar exon-length regions.

In any strain analyzed so far, six genes belonging to the fungal pheromone receptor sub-family of seven transmembrane domain receptors are found. These six genes (*bar3, bbr2*, *brl1, brl2, brl3, brl4*) showed the basidiomycete typical intron lengths with 50 bp. Regarding intron positions, *brl1* and *bar3* do not show introns in the 3′ part of the transcript. The 5′ region of *brl1, brl3, brl4*, *bar3* and *bbr2* is characterized by three stringed exon length regions of 110/113 bp, 67 bp and 138 bp (Figure 1). The genes coding for the four Brls in *S. commune* H4–8, *brl1* (ID 2638177)*, brl2* (ID 2704867), *brl3* (ID 2638261) and *brl4* (ID 2691538), are true orthologs of *S. cerevisiae* Ste3 [11], with three genes, *brl1, brl2* and *brl3*, located at the *B* mating-type locus, whereas *brl4* is localized separately.

From the conceptually translated protein sequences, seven transmembrane helices typical for GPRCs could be confirmed, and the Brls contain a long third cytoplasmic loop (more than 50% of the protein in case of Brl2) and carboxy terminus, like the Bar and Bbr pheromone receptors. They contain protein–protein interaction sites and polynucleotide/DNA binding sites, as well as putative domains, responsible for receptor recycling/degradation (for motif assignment, Figure S1).

**Figure 2:**
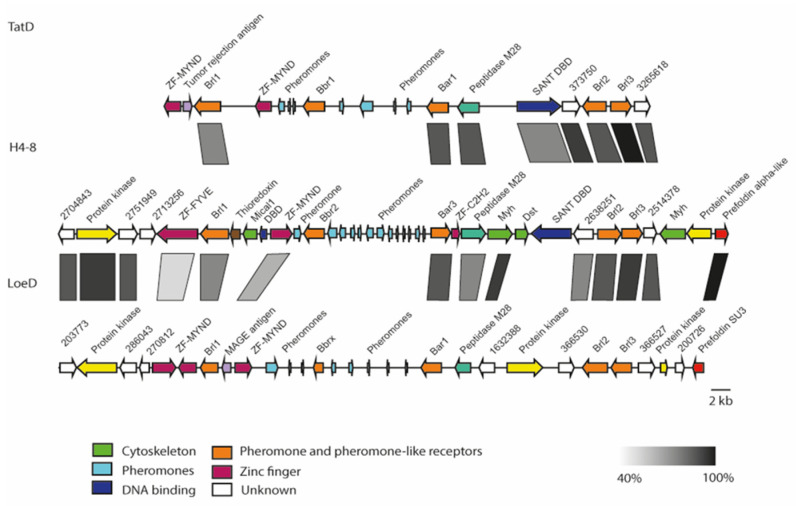
Chromosomal organization of *B* mating-type loci of *S. commune* strain H4–8 (scaffold 10), TatD (scaffold 230, 617, 960) and LoeD (scaffold 212, 284 and 343). The rectangle under the genes marks the highly syntenic regions (protein identity values are displayed in grey gradient 40–100%).

**Figure 3:**
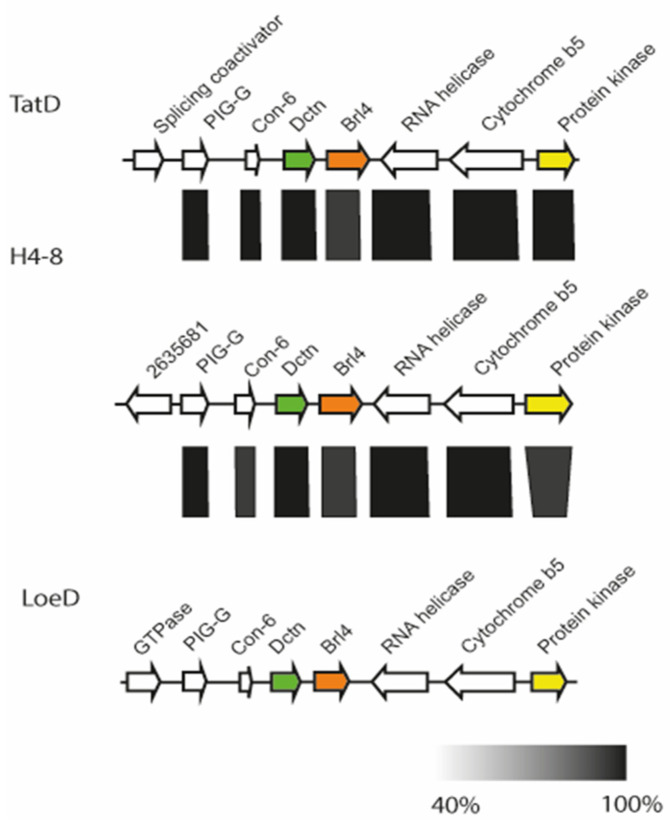
Chromosomal organization of the *brl4* surrounding in *S. commune* strain H4–8 (scaffold 8), TatD (scaffold 232) and LoeD (scaffold 60). The rectangle under the genes marks the highly syntenic regions (protein identity values are displayed in grey gradient 40–100%).

A comparison of the genomic loci from three available *S. commune* genome sequences showed high synteny (Figure 2). The *B* locus spans 32 kb, with a distance of 7 kb between the two mating-specific sub-loci *Bα* and *Bß*. The *brl1* genes are located upstream of the Bα pheromone receptor gene, *bar*, flanked by a zinc finger transcription factor (MYND, FYVE-type). A protein kinase and two hypothetical proteins, both located upstream of *brl1*, are highly conserved between the two strains *S. commune* H4–8 and *S. commune* LoeD. No pheromone genes are seen surrounding *brl1*. The genes *brl2* and *brl3*, which are located downstream of the *Bα* pheromone receptor *bar*, are flanked by two hypothetical genes, which show a high degree of similarity. All Ste3-like pheromone receptors and pheromone genes retained their position, while more distal genes were more variable, with genes related to the cytoskeleton interspersed. The gene *brl4* is not linked but again is flanked with genes similar in all strains, with a Con-6 domain protein close to *brl4* (Figure 3).

Alignments of pheromone receptor genes and conceptually translated proteins of *S. commune* H4–8 revealed that Bar3 and Bbr2 are more closely related to each other than either is related to any Brl protein (Table S3). Interestingly, Brl sequences are more closely related to Bar3 and Bbr2 than either is related to another Brl protein, indicating either that a divergence event separating Brl proteins from Bar3 and Bbr2 occurred before the two mating types evolved or that Bar3 and Bbr2 have undergone more recent gene conversion. The alignments also showed higher similarity for the N-terminal protein parts, suggesting a strong conservation of the N-terminal transmembrane domains and extra- or intracellular loops (Figure S2). A highly similar part of the C-terminal part of the second and the third extracellular loops extending into the adjacent transmembrane domains was found and highlighted in the 3D models and in the sequence alignment (Figure S3).

**Figure 4:**
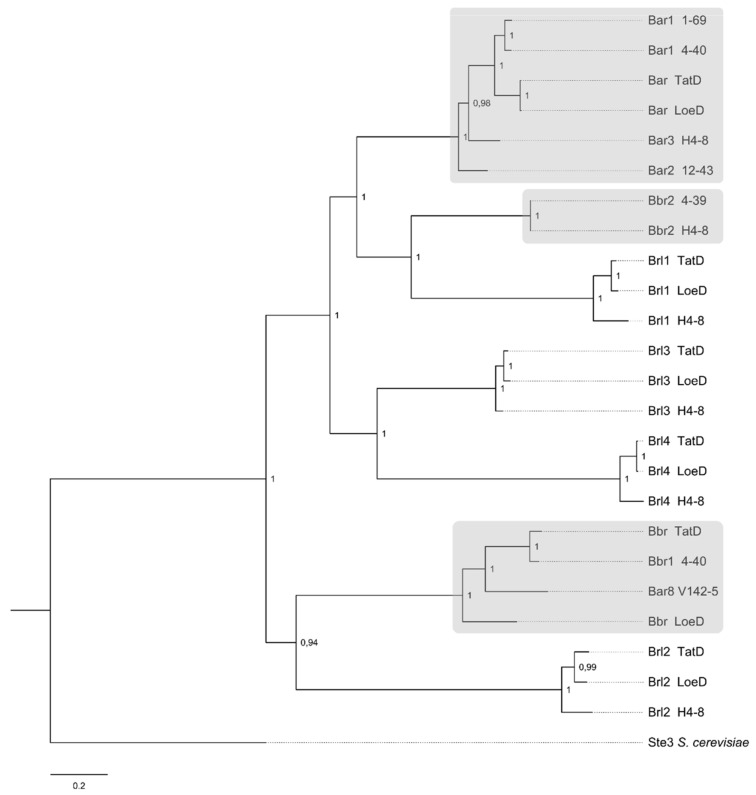
Phylogram of Ste3-like receptor proteins of *S. commune* strains TatD, LoeD and others. *S. cerevisiae* Ste3 protein served as outgroup (accession number P06783). Protein and DNA alignments were used in combination for calculation of the tree with Markov chain Monte Carlo method (Dayhoff model). Node labels indicate posterior probabilities. “Bar” and “Bbr” refer to true mating receptors (marked with grey); “Brl” refers to *B* receptor-like proteins. Used sequences and accession numbers (NCBI or JGI databases) of *S. commune* are: Bar1, strain 4–40 (X77949); Bar1, strain 1–69 (X94996); Bar, strain LoeD (ID 284719); Bar, strain TatD (ID 373755); Bar3, strain H4–8 (X3027970); Bar2, strain 12–43 (X91164); Brl1, strain LoeD (artificial); Brl1, strain TatD (ID 208691); Brl1, strain H4–8 (ID 112464); Bbr2, strain 4–39 (AF148501); Bbr2, strain H4–8 (EFI93340); Brl3, strain LoeD (ID 238914); Brl3, strain H4–8 (ID 258344); Brl3, strain TatD (ID 422121); Brl4, strain LoeD (ID 289019); Brl4, strain TatD (ID 373780); Brl4, strain H4–8 (ID 111749); Bbr, strain LoeD (ID 161825); Bar8, strain V142–5 (AAR99618); Bbr1, strain 4–40 (AAB41858); Bbr, strain TatD (ID 373756); Brl2, strain TatD (ID 215071); Brl2, strain LoeD (ID 168872); Brl2, strain H4–8 (ID 112482).

The phylogenetic clustering among the pheromone receptors showed *brl* gene products well separated from pheromone-recognizing mating-type receptors (clustering in Bα and Bβ receptors). The Brl1 group is most related to the known mating-type receptors Bbr2 of strain 4–39 and H4–8, while Brl2 clusters together with Bβ receptor sequences of TatD, 4–40 and LoeD. The Brl3 and Brl4 proteins are contained in a clade with Brl1 as well, but grouped separately. The Brl proteins of TatD and LoeD are always more closely related to each other than to the respective H4–8 protein (compare Figure 4). With this information, it is now possible to define four general clusters for the four *brl* genes, set apart from the two pheromone-recognizing receptors in *S. commune*. Thus, potentially different but overlapping roles for each of the Brl gene products can be envisioned. The analysis of expression and overexpression, therefore, was used to specifically address the regulation and role in cells of *S. commune*.

### 3.2. Transcriptome Expression Analysis of Brls

To evaluate signals for transcriptional control, the *brl* promotor sequences were analyzed using 1000 bp long regions before the start codons. Regulatory motifs in the first 500 to 600 bp of each promotor included a CTTCTTCCTCCCTTCTGCCTT motif. This is present in all promotor regions, including the pheromone-recognizing *bbr2* and *bar2.* This motif shows similarities to the yeast Tec1 transcription factor recognition site [47]. A second motif (consensus GACGCAaa) very similar to yeast’s Fhl1-binding motif [48] was found in the promoter regions of *bar3, bbr2, brl1, brl2* and *brl4* (Figure S4). To evaluate potentially similar regulation, a transcriptome analysis was performed.

Expression profiles of *brl* genes in wild-type 4–39 and 12–43 monokaryon, W22 × 12–43 dikaryon and several signal transduction mutants (*thn* mutant, *ras1*^G12V^ and Sccdc^G12V^) were determined by whole-genome microarrays (Figure S5). In order to compare the two monokaryon genetic backgrounds in a dikaryon, a co-isogenic line was used. From this, strain 4–39 was used for a back-cross to create a compatible mating type for crossings, strain W22. The high sequence similarity of *brl* genes in different strains facilitates their investigation within all stages on the array (Figure S5). The *brl3* gene showed differences in regulation between the two compared monokaryons (*S. commune* 12–43 and *S. commune* 4–39), suggesting a strain-specific function. Since *S. commune* 12–43 is auxotrophic for uracil, a cultivation-dependent regulation seems possible as well. For the dikaryon, a > 2-fold decrease in *brl1* and *brl2* was observed, while *brl3* was >2-fold upregulated in the dikaryon. This might indicate a specific function of Brl3 in the dikaryon.

Involvement of downstream signaling cascades into the analysis of differential expression will allow for the inclusion of those into function prediction. The constitutively active Ras signaling in a mutant *ras1*^G12V^ strain (12–43 vs. *ras1*^G12V^) showed dow-regulation of *brl3* by > 2-fold, which is in accordance with a function in mating-type signaling, as Ras activation is a result of pheromone binding to the cognate pheromone receptor. The gene *brl2* was > 2-fold downregulated in a *cdc42* mutant background (4–39 vs. Sccdc^G12V^), and *brl4* was > 2-fold downregulated in *thn* mutant (12–43 vs. *thn*). Since both Cdc42 and regulation through the regulator of G-protein signaling (RGS) are connected to the cytoskeleton and phenotypes in growth patterns, these data suggest involvement in dikaryon-specific growth types.

### 3.3. Overexpression of brl2, brl3 and brl4 Induces Asymmetrical Growth

**Figure 5:**
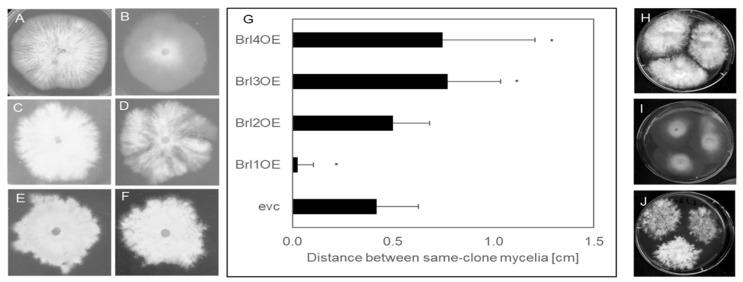
Morphology of empty vector control strain (**A**) and *brl* overexpressing strains (**B**–**F**). Strain Brl1 OE (**B**) showed a straight growth but produced less dense aerial hyphae, while strain brl2OE (**C**) grows more irregularly, forming dense aerial hyphae. The mycelia of *brl3* overexpressing strain Brl3–1OE (**D**) grows in a feathery manner with sectors growing faster than the rest of the mycelium, while Brl3–2OE (**E**) grows in a feathery manner but with dense aerial hyphae. Strain brl4OE (**F**) grows more irregularly when compared to the empty vector control strain forming dense aerial hyphae. Self-recognition of *brl* overexpressing strains (**G**–**J**). (**G**) Distance towards same clone mycelia was measured after 14 days from 5 biological replicates. (**H**) represents the empty vector control strain, while (**I**) shows the non-self-avoiding phenotype of the Brl1OE strain and the self-avoiding phenotype of Brl3OE (**J**) as an example. The Student’s *t*-test was used to determine the *P* value between control and transformants; (*) *p* < 0.005.

The genes *brl1, brl2, brl3* and *brl4* were overexpressed in *S. commune* T33 using the translation elongation factor *tef1* promotor. Monokaryotic colonies of the empty vector control (evc) and *brl* overexpressing strains (brl1OE, brl2OE, brl3OE and brl4OE) were analyzed for their phenotypes. *S. commune* brl1OE showed a straight growing phenotype with reduced aerial mycelia formation, while the remaining overexpressing strains grew irregularly, with bundles of hyphae or hyphal knots being formed locally (Figure 5). In addition, mycelial sectors growing faster than others were formed, resembling more the colony shape of a dikaryon (compare Figure S6). This phenotype was stronger on minimal than on complex medium or medium supplemented with wood chips.

Since overexpressing transformants in *S. commune* can show slightly different phenotypes depending on integration site, several transformants were screened for each *brl* gene. Only *S. commune* brl3OE yielded different phenotypes, with 30% of the colonies growing in a feathery manner with less aerial mycelia, while 70% grew more irregularly with more biomass. Quantitative PCR using RNA from representative transformants showed that in the feathery transformant (brl3–1OE), *brl3* was expressed at a lower level compared to transformants forming more aerial mycelia (brl3–2OE, Figure S7); brl3–1OE was selected for further experiments.

In all different overexpression transformants, a reduced growth rate without loss of colony biomass was observed (Figure S8). Thus, biomass formation and radial growth were assessed on sucrose and xylose in addition to the normally used glucose. Less biomass was observed for the *brl* overexpressing strains (Brl1OE, 0.8-fold only on xylose; Brl2OE, 0.7-fold on either sucrose or xylose; Brl3OE as well as Brl4OE, 0.6- and 0.5-fold reduction, respectively). The reduced biomass formation was accompanied by a reduced radial growth rate of Brl1OE, Brl3OE and Brl4OE on sucrose and xylose, which was not observed for BrlOE2. Microscopical analysis revealed that overexpression of *brl1* and *brl4* resulted in vacuole-rich, wide hyphae (suppl. Figure S9).

Dikaryotic-like growth of Brl2OE, Brl3OE and Brl4OE strains could enhance mating or fruiting body formation; thus, they were mated with compatible partners. The mating behavior was not different from the control and there was neither a lack of time nor a higher speed in mating (Figure S10). Clamps and fruiting bodies developed indistinguishably from wild type.

### 3.4. Brl2, Brl3 and Brl4 are Involved in Self-Recognition

When the *brl* overexpressing strains were cultivated with independent inocula growing towards each other on one plate, a self-avoidance phenotype became visible (Figure 5). This is in contrast to the wild type that will show intermingling mycelia without a gap or with only a minor gap between same-clone mycelia of up to 0.42 cm ± 0.21 cm. In the case of the *brl3* and *brl4* overexpressing mutants, the space between the mycelia was larger, with 0.77 ± 0.26 cm (*p* > 0.001) for Brl3OE and 0.75 ± 0.46 cm (*p* > 0.001) for Brl4OE. (Figure 5). In contrast, overexpression of *brl1* did not enhance self-recognition. Confrontation of Brl1OE, Brl2OE, Brl3OE and Brl4OE with each other did not result in any growth reduction (Figure S11).

### 3.5. Brl1 and brl4 Expression is Induced During Mating

**Figure 6:**
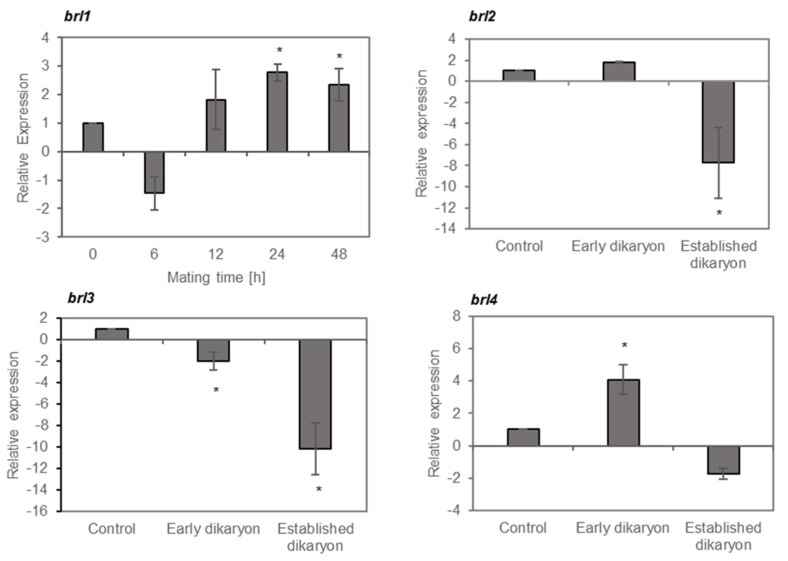
Expression of *brl1*, *brl2, brl3* and *brl4* in *S. commune* during mating (qRT-PCR). The expression of *brl1* was monitored over a time period of 48 h, while *brl2*, *brl3* and *brl4* were analyzed 24 h and 8 days after mating, corresponding to an early dikaryon and established dikaryon, respectively. The data were normalized to the expression in the monokaryon 4–39 (set to 1) and relatively quantified to three reference genes. (*) Significant differences ≥ 2-fold regulation.

The shared sequence identity between Bar3 and Bbr2 with pheromone receptor-like proteins suggested that Brls could have a function in pheromone sensing and signaling. Consistently, overexpression of *brl1* induces a flat growth with a reduced aerial mycelia formation also known for mutants in RGS signaling [49]. Thus, we investigated the pattern of *brl1* expression over a time period of 48 h during mating between 12–43 and 4–39 (Figure 6). Quantitative real-time expression analysis showed that *brl1* is downregulated 6 h after mating followed by a gradual increase up to 3-fold after 24 h (Figure 6). In contrast, overexpression of *brl2, brl3* and *brl4* led to an irregular colony growth with knot-like structures, which is typical for dikaryons. Thus, their expression was determined in the early dikaryon (24 h after mating) and in the established dikaryon (8 days after mating) corresponding to the growth conditions used for the microarray-based transcriptome analysis. Expression analysis showed that *brl2* and *brl3* were ≥2-fold repressed in the established dikaryon, suggesting a role of *brl2* and *brl*3 in vegetative growth. In comparison, *brl4* was ≥2-fold upregulated in the early dikaryon, indicating a temporal regulation of *brl4* in response to mating (see Figure 6). All four *brl* genes are expressed in strain V153–21 [50], a UV-radiated mutant, which is unable to mate due to a deletion of the *B* locus (Figure S12) suggesting a mating-independent function or an involvement beyond mating.

### 3.6. A Role in Clamp Fusion

**Figure 7:**
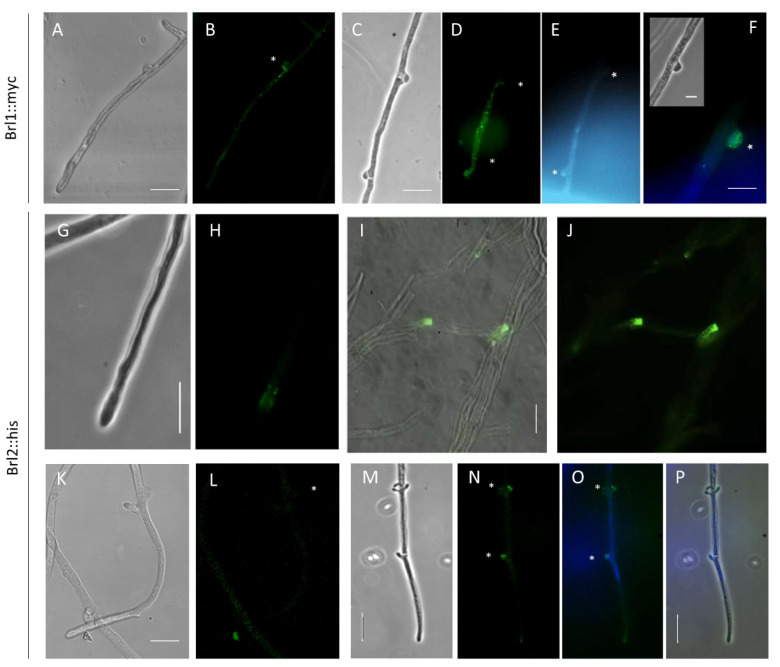
Localization of Brl1::myc in dikaryotic hyphae and Brl2::his in monokaryotic and dikaryotic hyphae. Localization of Myc-tagged Brl1 in dikaryon with wild-type 12–43 (**A**,**B**) and in Brl1::myc dikaryon with G11 (**C**–**F**) as shown by immunofluorescence staining with an anti-myc antibody 24 h after mating. Staining with DAPI (**E**) and merging of DAPI and FITC signal (**F**). Localization of Brl2::his in unmated hyphae (**G**–**J**). Localization of His-tagged Brl2 in dikaryon with wild-type 12–43 (**K**–**L**) and in Brl1::his dikaryon with G11 (**M**–**P**) as shown by immunofluorescence staining with an anti-his antibody 24 h after mating. Staining with DAPI (**O**) and merging of DAPI and FITC signal (**P**). Clamps are marked with asterisks. Bar represents 10 μm.

To visualize protein localization, Brl1 and Brl2 were tagged with C-terminal codon-optimized epitope tags, creating Brl1::myc and Brl2::his strains. For localization studies, the *brl* genes were under the control of their native promoter, ensuring natural expression levels, which resulted in transformants morphologically indistinguishable from wild-type strains. Brl1::myc was not detectable in monokaryons, and only a weak fluorescence could be observed in Brl1::myc dikaryons (Figure 7A,B) using a wild-type strain as a mate. However, in matings with a prolonged clamp cell fusion using the *S. commune* Bar2 receptor mutant G11 as a mate, Brl1 localization to pseudoclamps could be obtained (see Figure 7C–F).

The protein Brl2::his was detectable in monokaryons in vesicles and at the hyphal tips (Figure 7G–J, Figure S13). Brl2 was recruited to the membrane of hyphal tips when many hyphae were in close proximity to each other. To confirm the results, the transformant was crossed with *S. commune* G11, allowing a double staining of Brl2::his and Bar2::egfp. For both receptors, a membrane localization in clamps and pseudoclamps could be shown (Figure 7M–P and Figure S13). Moreover, an accumulation of both receptors in vesicles could be seen. Taken together, the specific subcellular localization indicates an involvement of Brl1 and Brl2 in clamp cell fusion. For Brl2, an additional role in hyphal communication is indicated.

## 4. Discussion

### 4.1. What Is the Difference: Comparing the Ligand-Recognizing Pheromone Receptors and Brls

The occurrence of four Brl genes in the first *Schizophyllum* genome had prompted the question of whether these are merely remnants of gene duplication events in this fungus known for its excessive number of mating specificities in nature [51,52]. Here, we compared three genomic sequences of *S. commune* and identified syntenic regions carrying pheromone-recognizing *Bα* and *Bß* and three *brl* receptor genes, as well as one copy for *brl4* placed outside the mating-type locus. The organization of the *B* mating-type locus, with fungal pheromone receptor genes, pheromone genes and neighboring pheromone receptor-like genes is conserved in many basidiomycetes [53]. Pheromone receptor-like genes have been found adjacent to the *B* mating-type loci in the genomes of other basidiomycetes as well, including *L. bicolor*, *F. velutipes*, *P. placenta* and *P. chrysosporium* [6,7,20]. An organization similar to *S. commune* was found in *S. lacrymans* [54] and *C. cinerea* [55], where two pheromone receptor-like genes are located next to the *B* locus. Since all four *brl* genes are expressed and share similarity and synteny, they must have a yet-unknown function.

The *brl* genes show a high sequence identity in closely related *S. commune* strains and a higher sequence divergence is shown in two more distantly related strains (TatD and LoeD). Still, they cluster in accordance with their placing within the extended mating-type locus in distinct groups, which is specifically well visible when including other fungal mating-type receptors and putative pheromone receptor-like proteins (for detailed phylogram, see [56]). Brl1 was closely related to *C. cinerea* Rcb2B6 (pheromone-recognizing receptor; [5]) and *F. velutipes* Ste3.2 (pheromone-recognizing receptor; [7]), while Brl3 clusters with the four pheromone receptor-like proteins of *F. velutipes* [7]. Brl2 was found in close association with Ste3.3 of *V. volvacea* and other pheromone-recognizing receptors from *L. edodes*, *P. placenta* and *T*. *vaccinum* [56]. The location of *brl4* outside of the mating-type locus may be indicative of a non-mating-specific function associated with the extensively high sequence conservation between the three *S. commune* strains. This specific assignment of related groups of Brls might indicate different functions.

### 4.2. Is There an Involvement of One or More Brls in Mating?

The close relation of Brls with pheromone-recognizing receptors of basidiomycetes suggests a function in mating. In response to mating and in an established dikaryon, qRT-PCR confirmed that the genes are not pseudogenes or silent products of receptor gene duplications. Overexpression of *brl* genes did not influence mating with compatible strains, and Brls are expressed in a mating-deficient strain carrying a large deletion covering the pheromone-recognizing receptors Bar and Bbr [10,50]. This evidence implies that the role of Brl proteins is not directly linked to mate recognition.

A conceivable function for Brls would be in heterodimerization with Bar and/or Bbr pheromone receptors. Mammalian GPCRs were found to undergo such oligomerization [57], thereby increasing the number of ligand binding sites. The new ligand binding pockets in such dimers or oligomers would fit nicely to the differential recognition of over 20 pheromones per mating-type receptor necessary for the discrimination of nine allelic specificities found for Bα and another nine for Bß recognition of non-self versus self pheromones [58]. The options for different protein–protein interactions of receptors can be outlined from interaction domains and dimerization signaling sequences present in all receptor sequences. For this function, direct pheromone recognition of the Brls on their own might not be possible, explaining earlier findings. The receptor proteins Brl1 and Brl2 were localized in the hyphae of *S. commune* transformants carrying the fusion proteins Brl1::myc-tag and Brl2::his-tag. Brl1 was found to localize in unfused clamps in a Δ*gap1* background, known for aberrant clamp formation [59], which might therefore elongate the time frame when Brl1 is expressed. At the same time, this was the place where the pheromone-recognizing Bar2 receptor was localized [11]. The successful detection of Brl1 in dikaryotic hyphae is correlated with the proposed function in mating and a potential contribution to self versus non-self recognition.

### 4.3. Functions Downstream of Direct Pheromone Interaction

The receptor protein Brl2::his-tag was also detected in clamps, pseudoclamps, in hyphae (most likely in vesicles used for receptor recycling and transport), at tips of the hyphae, at tips just after branching and near septa. The difference in spatial distribution between Brl1 and Brl2 points towards different functions of the two Brl proteins. The high abundance of Brl2::his-tag at tips, after branching and also where many hyphae were clumped together possibly indicates its importance during growth, determination of the growth direction and orientation of hyphae towards each other. Double staining of the fusion proteins Bar2::HA and Brl2::his-tag revealed the localization of both in unfused clamps of the dikaryon. Hence, Brl1 and potentially also Brl2 are connected to mating and sexual development with clamp fusion.

Consistently, promotor analysis put the *brl* genes in the context of mating and filamentous growth, identifying a prominent motif similar to the yeast Tec1 transcription factor binding site. Tec1 is involved in the transcription of filamentation genes and forms a complex with Ste12, which is a transcription factor regulating mating-dependent gene expression [47]. The pheromone-recognizing receptor gene *bar2* [11], *brl1* and *brl4* showed a similar pattern, with upregulation shortly after mating in the early dikaryon. Thus, different roles for the single *B*-like receptors may be expected, and those functions likely are related to processes induced by mating interactions. Thus, we propose for Brl2 a function after mating and in clamp fusion.

A different regulation of *brl2* and *brl3* was seen in RasGap1 and Cdc42 mutant background. This rather places them into signaling pathways influencing cytoskeleton formation via Cdc42, cAMP and MAPK cascades, mainly influencing fruiting body formation and pheromone response [60]. The transcription factor Fst4 seems to influence the expression of *brl*2, which was significantly induced in the Δ*fst4*Δ*fst4* dikaryon. In contrast, the pheromone-recognizing *bar3* and *bbr2* genes were stably expressed [61]. A motif similar to the Fhl1-binding motif of yeast was found in *bbr2* and with a lower similarity in *bar2, brl1, brl2* and *brl4.* The transcription factor Fhl1 is known to bind Rap1, and the complex then regulates the expression of several genes and is involved in cell wall maintenance [48], while the localization of Brl2 near the hyphal tip indicated a role in hyphal growth.

### 4.4. Self-Recognition through Brls

Both *brl2* and *brl3* did not change their expression in the early dikaryon but instead were repressed in the established dikaryon.

This is in line with the differential expression pattern of *brl* genes in the lifecycle. The genes coding for dynamitin and Con-6 are located as direct neighbors to *brl4,* and an induction of *con-6* was confirmed in mating interactions, mostly in W22 × 12–43 but also in the signal transduction mutants of *S. commune* [11,60]. The upregulation of *con-6* indicates an involvement in dikaryon and mushroom formation, similar to *N. crassa* Con proteins functioning in asexual spore development [62].

We propose a function of Brl3 and Brl4 in hyphal self-sensing and -avoidance based on the phenotypes observed in overexpression analyses. The receptors, however, so far are orphans. Potential ligands could be self-pheromones that after recognition do not lead to hyphal attraction but rather induce hyphae to grow away from the pheromone source. This would create the observed self-avoidance phenotype and be in line with the asymmetrical, dikaryon-like growth of *brl3* overexpressing mutants and the downregulation of *brl3* in the established dikaryon. Thus, self-pheromones would act as auto-inhibitors, which had been postulated earlier to explain feathery hyphal growth in dikaryons [63].

The function of an inhibitor secreted by the mycelium would slowly diffuse, producing an inhibitory front. Hyphae ahead of this front are not inhibited, causing the leading growth and finally asymmetry of the colony. Aside from self-pheromones acting as auto-inhibitors, other ligands might be involved. The volatile compounds 1-octen-3-ol and 3-octanone, which control conidia germination of *A. nidulans* [64], are known to inhibit the growth of other fungi [65]. In addition, small secreted proteins common to fungi are suggested to play a role in development and fruiting [66]. A study identified 10 putative small secreted proteins in the genome of *S. commune,* which might function as auto-inhibitory substances [61]. Our results indicate that Brl3 and Brl4 are involved in sensing this auto-inhibitor, creating the typical asymmetrical colony.

As to intracellular signaling involved in response to an auto-inhibitor, the transcription factors *hom2* and *bri1* have been reported to be involved in mycelial growth, and dikaryons homozygous for Δ*hom2* and Δ*bri1* form symmetrical colonies under conditions that would usually show asymmetrical growth, again postulated to be exerted by regulation through a postulated auto-inhibitor. Inactivation of these transcription factors abolished early stages of fruiting body formation [61].

In contrast to the symmetrically growing monokaryons, dikaryons of *S. commune* form asymmetrical colonies, which are able to produce fruiting bodies only in response to light. In the dark, dikaryons grow symmetrically and mushroom formation is abolished. It could be shown that the blue light receptor genes *wc-1* and *wc-2* are involved this light-stimulated development [2]. Hence, yet another layer of regulation is imposed on self-avoidance.

### 4.5. Different Functions of the Four Brls Have Evolved in This Highly Diversified Tetrapolar Mating System

Since Brls are present in multiple basidiomycete genomes, a functional characterization of the loci seems appropriate to unravel the puzzle of why receptors not involved in pheromone recognition should have been conserved in evolution. The comparison of the sequences of all four Brls with Bar3 and Bbr2 of *S. commune* H4–8 revealed the highest similarities between the true mating-type receptors Bar3 and Bbr2 (29%, amino acid level) [67]. The most unrelated receptor was Brl2, with only 17% identity to Bbr2 or less to the other sequences.

All Brls share higher identity with the pheromone-recognizing mating-type receptors than with each other, and the resulting phylogenetic tree indicates that they developed from several independent receptor duplication events. The higher conservation of the N-terminus of Brl proteins can be explained by the limited sequence variation of the transmembrane helices involving only a few amino acids to fulfill the requirements to form a transmembrane, α-helical structure [68]. This part has a potential influence on ligand/pheromone recognition and may be under high evolutionary pressure to be conserved. Mutations of this region caused new receptor phenotypes in *S. commune* [12,13]. Here, Brls are strikingly similar, which supports their diversified roles not directly associated with pheromones and their specific recognition.

We could establish that Brl1 is involved in mating and localized in pseudoclamps, while the localization of Brl2 near the hyphal tip indicated a role in hyphal growth. In contrast, *brl3* and *brl4* control dikaryotic asymmetrical growth.

Overexpression of *brl1* led to a flat phenotype with a reduced vegetative growth rate concerning colony diameter. However, biomass formation was not significantly reduced, indicating a compensation of the reduced aerial hyphal formation by substrate mycelium. The colony contained wide, vacuole-rich hyphae suggestive of autophagy and cell permeabilization that can be observed in fungal interactions, as well as in autophagy [69,70].

In contrast to the more similar N-termini, Brls’ C-termini were long and more diverse. The C-terminus is the target for several intracellular protein–protein interactions for signal transduction. The Brls share specifically long intracellular C-termini, which were more variable, including various protein-binding sequences and kinase-binding sites suggesting a complex signaling network. All in all, our study thus could infer different roles from phylogenetic, expression and overexpression studies that make these members of the class of fungal pheromone receptors an interesting target for different intracellular roles connected to mating in tetrapolar basidiomycetes.

## Data Availability

All data are available at JGI (*S. commune* genome sequences) or GEO omnibus (http://www.ncbi.nlm.nih.gov/geo/query/acc.cgi?accGSE26401 (accessed on 20 May 2021)).

## Supplementary Materials

The following are available online at https://www.mdpi.com/article/10.3390/jof7050399/s1, Table S1: *S. commune* wild-type and mutant strains used in this study, Table S2: Primers used in this study, Figure S1: Protein characteristics for Brls, Bar3 and Bbr2 of *S. commune* H4-8 and Ste3 of *S. cerevisiae*, Table S3: DNA and protein sequence identities, Figure S2: Alignment of N-terminal amino acid sequences of receptors from *S. commune,* Figure S3: 3D models of receptor proteins, Figure S4: Promoter analyses, Figure S5: Expression analysis of *S. commune brl* genes in wild-type and mutant strains, Table S4: Sequence identity of *brl* genes in several *S. commune* strains compared to the respectively sequence of *S. commune* H4-8, Figure S6: Morphology of dikaryotic *S. commune* strain 12-43x4-39, Figure S7: Absolute quantification by qRT-PCR of *brl3* expression, Figure S8: Growth (A-C) and biomass (D-F) of the empty vector control strain and *brl* overexpression strains using glucose (A, D), sucrose (B, E), and xylose (C, F) as carbon source, (G) shows the formed biomass/cm^2^ on glucose, sucrose and xylose, Figure S9: *S. commune* brl1OE (A) and brl4OE (B) form vacuole-rich wide hyphae, Figure S10: Mating interactions of T33, evc and *brl*-overexpressing strains with the compatible partner 12-43, Figure S11: Confrontation of Brl1OE, Brl2OE, Brl3OE and Brl4OE with each other did not result in any significant growth reduction, Figure S12: Expression of *brl1, brl2, brl3* and *brl4* in *S. commune* V153-21 (B_null_), Figure S13. Localization of Brl2::his (A-C) and Bar2::egfp in dikaryon.
