# Supplementary material for: What Role Might Non-Mating Receptors Play in Schizophyllum commune?
Source: J Fungi (Basel). 2021 May 20;7(5):399. doi: 10.3390/jof7050399 (PMC8161036; doi:10.3390/jof7050399)
Supplement: Supplementary file 1 [file jof-07-00399-s001.zip › jof-1186064-supplementary.pdf]

Supplemental Material

Table S1: *S. commune* wild-type and mutant strains used in this study.

Strain	Mating type	Genotype	Origin
12-43	$A_{3,5}$ $B_{2,2}$	*ura*$^-$	JMRC, FSU 3214
4-39	$A_{4,6}$ $B_{3,2}$		JMRC, FSU 2896
T33	$A_{3,1}$ $B_{4,7}$	*trp*$^-$; *ura*$^-$	JRMC, FSU 3446
evc	$A_{3,1}$ $B_{4,7}$	*trp*$^-$; *ura*$^+$	This study
Brl1OE	$A_{3,1}$ $B_{4,7}$	*trp*$^-$; *tef*^{P}::*brl1*	This study
Brl2OE	$A_{3,1}$ $B_{4,7}$	*trp*$^-$; *ble*; *tef*^{P}::*brl2*	This study
Brl3OE	$A_{3,1}$ $B_{4,7}$	*trp*$^-$; *tef*^{P}::*brl3*	This study
Brl4OE	$A_{3,1}$ $B_{4,7}$	*trp*$^-$; *tef*^{P}::*brl4*	This study
Brl1-myc	$A_{3,1}$ $B_{4,7}$	*trp*$^-$; *ura*$^-$; *ble*$^+$; *brl1*-myc	This study
Brl2-His	$A_{3,1}$ $B_{4,7}$	*trp*$^-$; *ura*$^-$; *ble*$^+$; *brl2*-His	This study
G11	$A_{3,5}$ Δ*matB*::*bar2*Δ*Pst*I-HA-*gfp;*	*trp*$^-$; *bar2*-HA-GFP	JMRC, FSU
V153-21	$A_{3,5}$ B_{null}	*trp*$^-$	JMRC, FSU

JMRC: Jena Microbial Resource Collection, Jena, Germany

Table S2: Primers used in this study.

Primer name	Sequence (5' to 3')
act-N1	GTCCGCCCTCGAGAAGAGTTA
act-N2	TTGTACGTCGTCTCGTGGATA
tef-N3	AGCTTGGCAAGGGTTCCTTCA
tef-N4	AACTTCCAGAGGGCGATATCA
ubi-for	GAAGGAGTACGATGCGAAGG
ubi-rev	TCCTCCTCTGCCTTCTTGC
brl1-for	GAGCTGTGCAGGACGCGAAGC
brl1-rev	ACGGCGGGATCACATTGACA
brl2-for	CACCGAAGCCGCCCAAACCTGCC
brl2-rev	TACGAAGGTCGTGGAGGCCG
brl3-for	ACCTGAACTTCTCGCACGTT
brl3-rev	GCACAGACGAAGATGAACCA
brl4-for	TCTCGCTACCTGGATCTCGT
brl4-rev	AACATCCACCTCGACACCTC
brl1_for_prom	GCTTGAACGCCGGGTAGTCC
brl1_rev_myc	TTAGAGATCCTCTTCTGAGATGAGCTTCTGCTCTACCTCTTCGACCTCGCGCT
brl2_for_prom	GATATCGCAATCGTCATCGC
brl2_rev_His	TCAGTGGTGATGGTGATGGTGGAACGCCTGCCCAAAAAC
ura2-for	GACCTGTTCCCCTTTCTTAGC
ura2-rev	TCAGCCGGATGCTGGACTA
ble-for	TCTAGAGATCTGACGTGCATTGTG
ble-rev	TCTAGACCAGCTTGCTCCAAAGAG
pRS_tef_fw	GGCCCCCCCTCGAGGTCGACGGTATCGATAAGGCGGCGGCTCCGGCTGGGGCGC
tef_brl1_rev	GAGGGGATACGTGGGGTCGTTCGAGAGCATTTTGAGTGTTTTCTAAGTGAG
tef_brl1_fw	ACTGACAATCTCACTTAGAAAACACTCAAAATGCTCTCGAACGACCCCACG
brl1_rev_in	CGAGTCCGGCCAGGCCTTCTCC
brl1_for_in	CGGCCTAGCTGGCGAGGCGAGC
brl1_pRS	CGCGGTGGCGGCCGCTCTAGAACTAGTGGACGCCAAAGCGTGAGGAGATCG
tef_brl2_rev	AAATGAGGATACAGGGAATTCGGCGCGCATTTTGAGTGTTTTCTAAGTGAG
tef_brl2_fw	ACTGACAATCTCACTTAGAAAACACTCAAAATGCGCGCCGAATTCCCTGTATC
brl2_pRS	CGCGGTGGCGGCCGCTCTAGAACTAGTGGAGCCACACTGGCATGATGCGC
tef_brl3_rev	CATGGAGAAGACCCAGTTGGGATACGACATTTTGAGTGTTTTCTAAGTGAG
tef_brl3_fw	ACTGACAATCTCACTTAGAAAACACTCAAAATGTCGTATCCCAACTGGGTC
brl3_pRS_rev	CGCGGTGGCGGCCGCTCTAGAACTAGTGGAGAGATGAAGGTGGAGGAAAC
tef_brl4_rev	GAACGCAAACGCGACGTTCGCTGCCGACATTTTGAGTGTTTTCTAAGTGAG
tef_brl4_fw	ACTGACAATCTCACTTAGAAAACACTCAAAATGTCGGCAGCGAACGTCGC
brl4_pRS_rev	CGCGGTGGCGGCCGCTCTAGAACTAGTGGAGCAGACAGGCTGCACCAACG

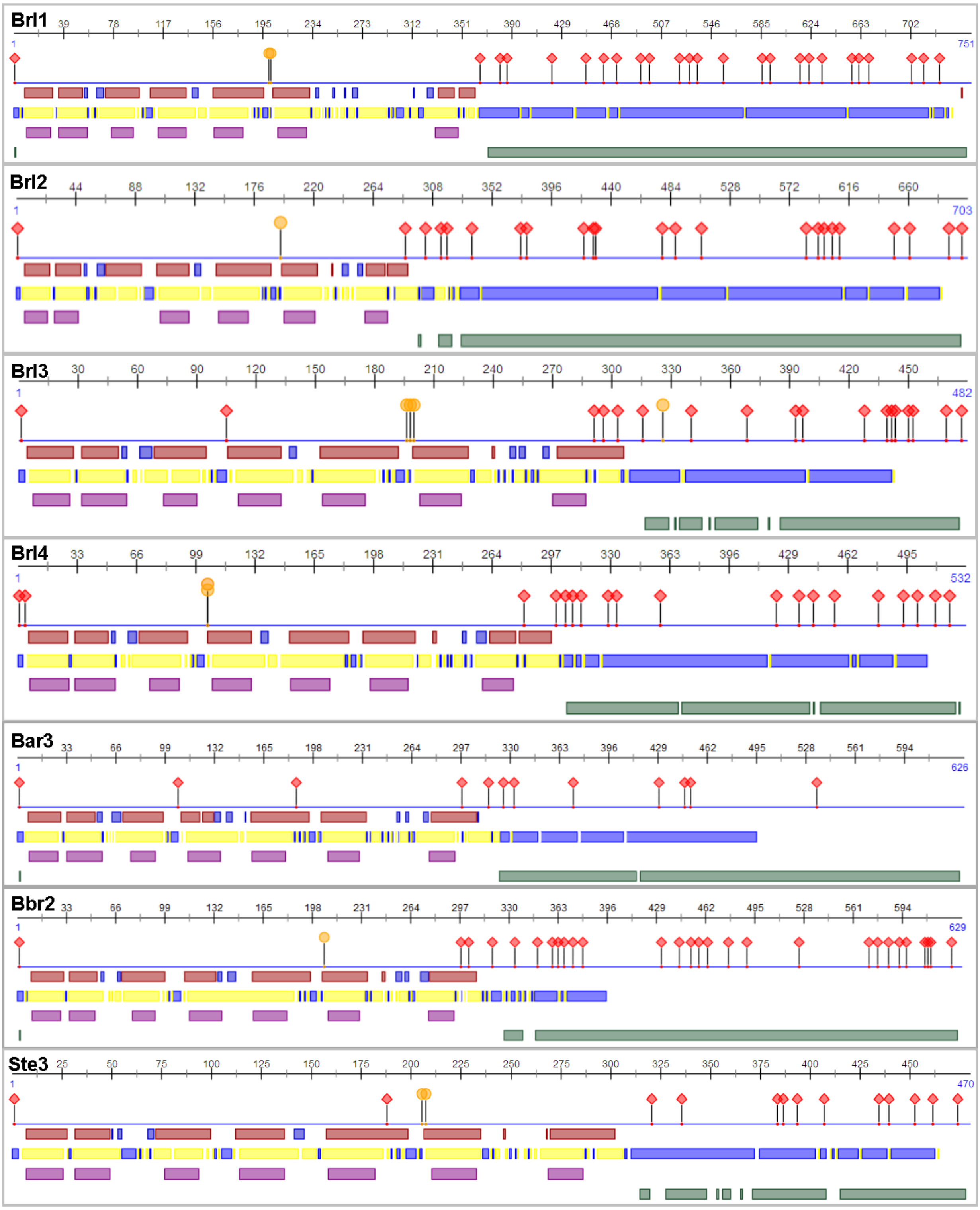

Figure S1: Protein characteristics for Brls, Bar3 and Bbr2 of *S. commune* H4-8 and Ste3 of *S. cerevisiae*. Amino acid positions are indicated, red rhombi show protein-protein-interaction site (signal peptide cleavage site and C-terminus effected, REPROFSEC prediction), orange circles for polynucleotide/DNA binding sites, auburn boxes show helical structures. Buried (yellow; not able to interact with the outside), and exposed” structures (blue, PROFAcc prediction are indicated. Purple boxes indicate helical transmembrane regions (TMSEC prediction). The green bar indicates a disordered region (MD prediction).

Table S3: DNA and protein sequence identities.

**Sequence**	**Brl1**	**Brl3**	**Bar3**	**Bbr2**	**Brl4**	**Brl2**
brl1	ID	0.14	0.18	0.27	0.16	0.11
brl3	*0.31*	ID	0.25	0.21	0.19	0.1
bar3	*0.37*	*0.43*	ID	0.29	0.2	0.12
bbr2	*0.39*	*0.42*	*0.48*	ID	0.2	0.17
brl4	*0.28*	*0.35*	*0.33*	*0.31*	ID	0.14
brl2	*0.32*	*0.27*	*0.31*	*0.32*	*0.27*	ID
**Sequence**	**Bar3 N'**	**Bbr2 N'**	**Brl1 N'**	**Brl3 N'**	**Brl4 N'**	**Brl2 N'**
Bar3 N'	ID	0.56	0.27	0.38	0.3	0.18
Bbr2 N'	*0.56*	ID	0.36	0.36	0.29	0.21
Brl1 N'	*0.27*	*0.36*	ID	0.27	0.23	0.2
Brl3 N'	*0.38*	*0.36*	*0.27*	ID	0.37	0.22
Brl4 N'	*0.3*	*0.29*	*0.23*	*0.37*	ID	0.25
Brl2 N'	*0.18*	*0.21*	*0.2*	*0.22*	*0.25*	ID
**Sequence**	**Bar3 C'**	**Bbr2 C'**	**Brl1 C'**	**Brl3 C'**	**Brl2 C'**	**Brl4 C'**
Bar3 C'	ID	0.22	0.07	0.1	0.11	0.12
Bbr2 C'	*0.22*	ID	0.15	0.09	0.14	0.09
Brl1 C'	*0.07*	*0.15*	ID	0.05	0.06	0.09
Brl3 C'	*0.1*	*0.09*	*0.05*	ID	0.05	0.09
Brl2 C'	*0.11*	*0.14*	*0.06*	*0.05*	ID	0.06
Brl4 C'	*0.12*	*0.09*	*0.09*	*0.09*	*0.06*	ID

DNA identities are on the left (italics); protein on the right site. 1 = 100 %.

```
Brl1_LoeD_artif MFSNDPTYPLFPILAFLSSVLALLPVPFTLQVWNTGACALAVWTATACLLSFINSVVWAG
Brl1_TatD_ID208 MLSNDPTYPLFPILAFLSSVLALLPVPFALQVWNTGACALAAWTATACLLSFINSVVWAG
Brl1_H4-8_ID263 MLSNDPTYPLFPILAFLSSVLALLPVPFTLQVWNTGACALAAWAATACLLSFINSVVWSG
Brl3_LoeD_ID238 MSYPNWVFSMF---MVFGIIMTLLPLPWHLEAWNTGTCLYMLWVAVACLCQLVNSIVWSG
Brl3_TatD_ID422 MSYPNWVFSMF---MVFGIIMTLLPLPWHLEAWNTGTCLYMLWVAVACLCQLVNSIVWSG
Brl3_H4-8_ID263 ---------MF---MVFGIIMTLLPLPWHLEAWNTGTCLYMLWVAVACLCQLVNSIVWSG
Brl4_LoeD_ID289 MSAANVAFAFF---AFVTFILVLVPLPWHLQAWNSGVCIYIGWVALGSIILYVDAVVWNG
Brl4_TatD_ID373 MSAANVAFAFF---AFVTFILVLVPLPWHLQAWNSGVCIYIGWVALGSIILYVDAVVWNG
Brl4_H4-8_ID269 MSAANVAFAFF---AFVTFILVLVPLPWHLQAWNSGVCIYIGWVAVGSIILYVDAVVWNG
Brl2_TatD_ID215 MRAE---LLVF---SFLSVCILLVALPFKRVRRDIPSTCLLLWLLAWNVIHAVNAVMWAG
Brl2_LoeD_ID168 MRAE---FPVF---SFLSVCILFVALPFKRVRRDIPSTCLLLWLLALNVIHAVNAVMWAG
Brl2_H4-8_ID270 MRAE---FPVS---SFLSVSIILVALPFKRVRRDIPFTCLLLWLLAWNVIHAVNAVMWAG
                         .     ..   : ::.:*:     :        *     :   :::::* *

Brl1_LoeD_artif NVMDTVPVWCDISSKLILGASIGIPASGLCISHRLWKISSTRHVPETRRETLLSIGIDLS
Brl1_TatD_ID208 NVMDTVPVWCDISSKLILGASIGIPASGLCISHRLWKIFSTRHVPETRREVLLSIGIDLS
Brl1_H4-8_ID263 NVMNTVPAWCDISSKLILGASIGIPASGLCISHRLWKVTSMRLVPETRREILFAIAIDLA
Brl3_LoeD_ID238 NAIDWAPVWCDISARVIIGTSVAIPAASLVINRRLYYIATVRTVTKSKPERRREVMMDLL
Brl3_TatD_ID422 NAIDWAPVWCDISARVIIGTSVAIPAASLVINRRLYYIATVRTVTKSRPERRREVMMDLL
Brl3_H4-8_ID263 NAIDWAPVWCDISARVIIGTSVAIPAASLVINRRLYYIATVRTVTKSKPERRREVMMDLL
Brl4_LoeD_ID289 NIIDPMPAWCDFSGRIMVAVPYGLEAATLCIQRRLYHIVRSSDVS-SKTSKRRWVIVDVL
Brl4_TatD_ID373 NIIDPMPAWCDFSGRIMVAVPYGLEAATLCIQRRLYHIVRSSDVS-SKTSKRRWVIVDVL
Brl4_H4-8_ID269 NINDPIPAWCDFSGRIMVAVPYGLEAATLCIQRRLYHIVRSSDVS-SKTSKRHWVIVDVL
Brl2_TatD_ID215 NADVRALPWCDLVTKVMLAALMGVAGCALCMLRVLEFLASARNP--GPNARRNTVSIEVL
Brl2_LoeD_ID168 NADVRALPWCDLVTKVMLAALMGVAGCALCMLRVLEFLASARNP--GPNARRNTVSIEVL
Brl2_H4-8_ID270 NAGVRALAWCDLVTKVMLAALMGVAGCALCMLRVLEFLASARNP--GPNSRRNTVSIEIL
                *       ***:  ::::..  .: .. * : : *  :                :  :::

Brl1_LoeD_artif LSAGTPLLVMLLHIIVQDHRSTILEDFGCHAAMPNTLLSYFLVLSWPLGLGLASLVYAVL
Brl1_TatD_ID208 LSAGIPLLVMLLHIIVQDHRFTILEGFGCHAAMPNTLLSYFLVLSWPLGLGLASLVYAVL
Brl1_H4-8_ID263 LSAGVPLFVMLLHIIVQSQRFTILEDIGCQAAIPNTLLSYFLVLSWPLGLGLASLIYAVL
Brl3_LoeD_ID238 IGMGLPVLVMILQYIPQGHRYDIYEDIGCYPATYNTWVAIVCVTGWPIAIGCVSAVYCML
Brl3_TatD_ID422 IGMGLPVLVMILQYIPQGHRYDIYEDIGCYPATYNTWVAIVCVTGWPIAIGCVSAVYCML
Brl3_H4-8_ID263 IGMGLPVLVMILQYIPQGHRYDIYEDIGCYPATYNTWVAIVCVTGWPIAIGCVSAIYCML
Brl4_LoeD_ID289 IAVVLPCVLMGLQYIVQGHRYDIYENYGCMLNTYMTGPAVLILRGPPIILGVIAGVYCVL
Brl4_TatD_ID373 IAVVLPCVLMGLQYIVQGHRYDIYENYGCMLNTYMTGPAVLILRGPPIILGVIAGVYCVL
Brl4_H4-8_ID269 IAVVLPCVLMGLQYIVQGHRYDIYENYGCMLNTYMTGPAVLILRGPPIILGVIAGVYCVL
Brl2_TatD_ID215 FCIGIPVLYMLLHFIVQDQRFTIAADIGCVASIHPSSPSLILMWLPPAIECVAAFALSCI
Brl2_LoeD_ID168 FCIVIPVLYMLLHFIVQDQRFAIAADIGCVASIHPSSPSLILMWLPPAIECVAAFALSCI
Brl2_H4-8_ID270 FCIVIPVLYMLLHFIVQDQRFSIAADIGCVASIHPSSPSLILMWLPPAIECVAAFALSCI
                :    * . * *: * *.:*  *  . **      :  : . :   *      :   . :

Brl1_LoeD_artif TLRLSYV-HRLEFAHALAKLGTLRTSLCLRLVLLALVAFTLSVVSSSLDIAALARSGPLI
Brl1_TatD_ID208 TLRLSYV-HRLEFDHALAKLGALRTSLCLRLVLLALVAFTLSVVSSSLNIAALARSGPLI
Brl1_H4-8_ID263 TLRLSYV-HRLEFDHALAKQGTLRTALCLRLVLLALVAFTVSIVSSSLNIAALARSGPLI
Brl3_LoeD_ID238 SIYNFNK-RRRHFNEYLAAHSNLTANRYFRLMCLAAIEICLTIPIGSFGLYMNTQFSEMQ
Brl3_TatD_ID422 SIYNFNK-RRRHFNEYLAAHSNLTANRYFRLMCLAAIEICLTIPIGSFGLYMNTQFSEMQ
Brl3_H4-8_ID263 SIYNFNK-RRRHFNEYLAAHSNLTANRYFRLMCLAAIEICLTIPIGSFGLYLNTQFSEMQ
Brl4_LoeD_ID289 LIRIFTQ-KRRRANT-----GPISRGICLRLIMLSTATLLFTVSLNLYTLIEDTRYG-LA
Brl4_TatD_ID373 LIRIFTQ-KRRRANT-----GPISRGICLRLIMLSTATLLFTVSLNLYTLIEDTRYG-LA
Brl4_H4-8_ID269 LIRIFTQ-KRRRANT-----GPMSRGICLRLIMLSTATLLFTVSLNLYTLIEDTRYG-LA
Brl2_TatD_ID215 AVHHATRLPSTLLDEHLSTRTPISSGLLLRNLCFASTACSLVVIAVLFGVFSPT----VV
Brl2_LoeD_ID168 AVHHATRVPSTLLDEHLSTRTPISSGLLLRNLCFASTACSLVVIAILFGVFSPT----VV
Brl2_H4-8_ID270 AVHHATRLPSTLLDEHLSTRTPISSGLLLRNLCFASTACSLVVIAVLFGVFSPT----VV
                 :                    :     :* : ::     . :      :   :    :
```

Figure S2: Alignment of N-terminal amino acid sequences of receptors from *S. commune*. Conserved regions are visible in the lowest rows, if these conserved parts are within the extracellular loops they are highlighted by boxes.

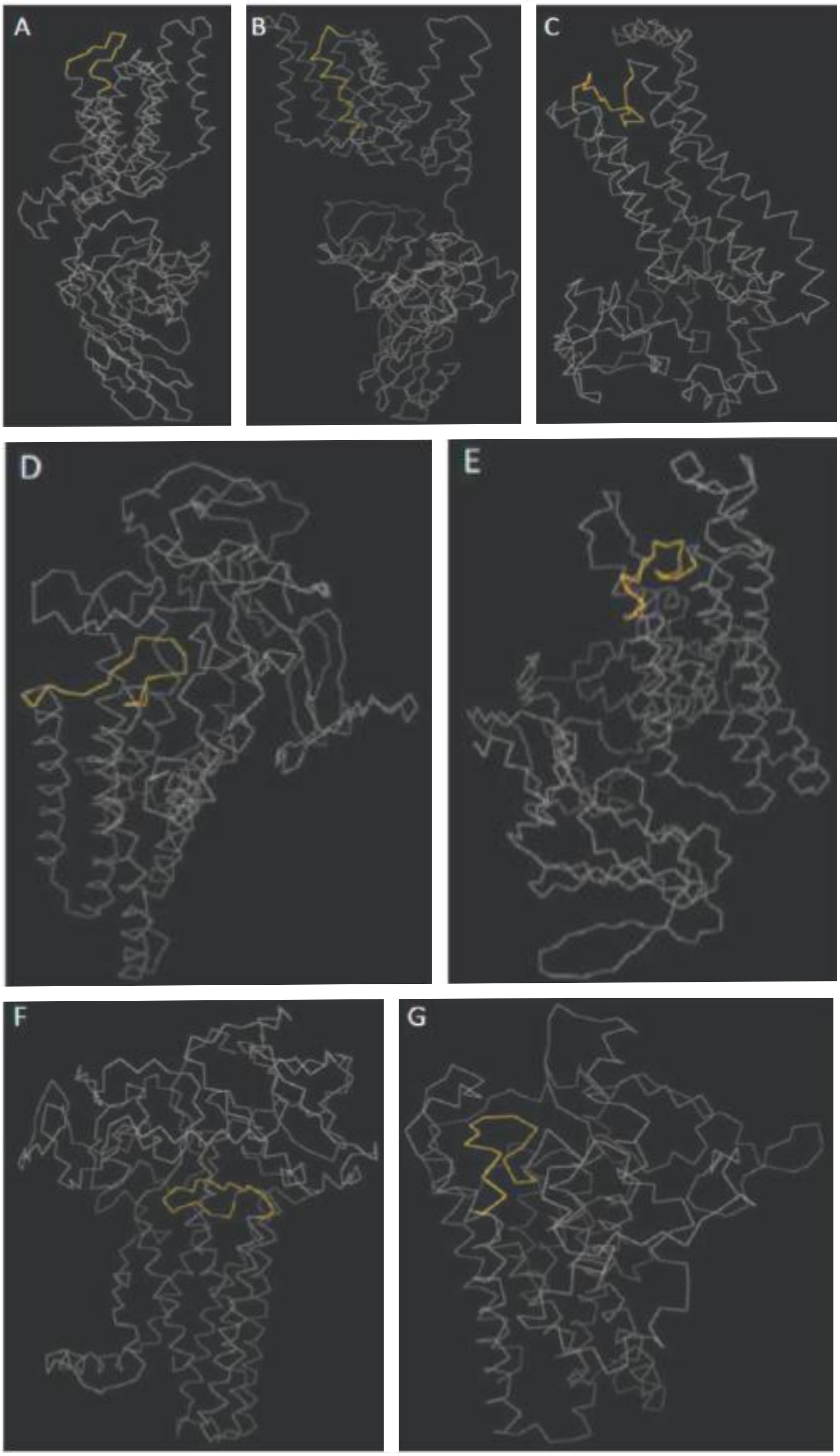

Figure S3: 3D models of receptor proteins. Protein secondary structures were modelled with ITASSER-GPCR and the model with highest c-score was taken for visualization. Grey line shows protein backbone e.g. in A with helical transmembrane area at top, intracellular C-terminus at bottom. Conserved extracellular loop is marked in yellow. **A** – Brl1. **B** – Brl2. **C** – Brl3. **D** – Brl4. **E** – Bar3. **F** – Bbr2. **G** – Ste3 of *S. cerevisiae*.

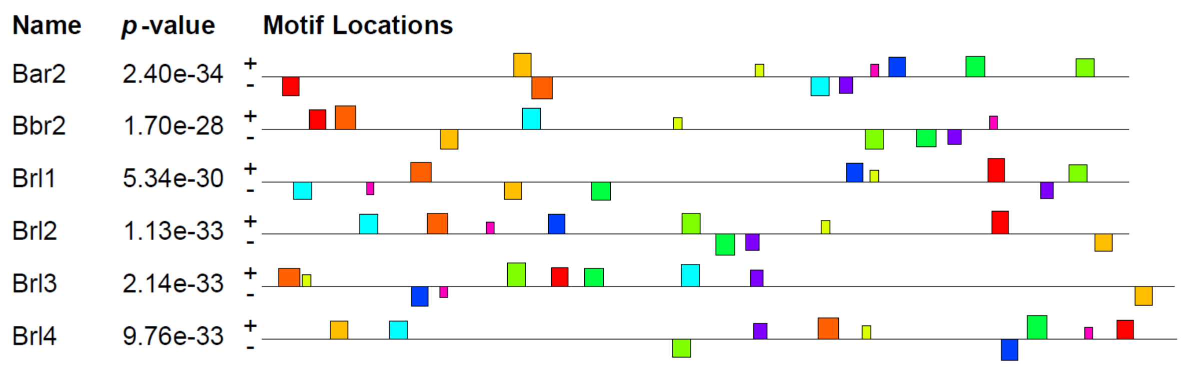

Motif	Symbol	Motif Consensus
1.		CTTCTTCCTCCCTTCTGCCTT
2.		TTGTTSGNCRTBCTCWGCCGC
3.		CTNTYMCDCCGTWMYTCMTCG
4.		GTGAWGGSBAATGKT
5.		RKGTGKGSGAWKNSGARGKA
6.		AACTSGKGMWGCCTYCCTNGCG
7.		GCAHCTCRTTKTSCSCANA
8.		ATTGTYCTC
9.		MGACKYCTRCASACGMMTATCBAT
10.		TVTACTTTAC

	Motif Consensus GACGCAaa
bar2	1
bbr2	3
brl1	2
brl2	3
brl3	0
brl4	3

Figure S4: Promoter analyses. Motifs found in the promoter regions of *brl1, brl2, brl3, brl4, bar3* and *bbr2* by MEME and MAST. Motif1 is similar to yeast Tec1 binding site. A motif similar to yeast's Fhl1-binding motif was found by manual sequence inspection in the promoter regions of *bar2, bbr2, brl1*, *brl2* and *brl4*

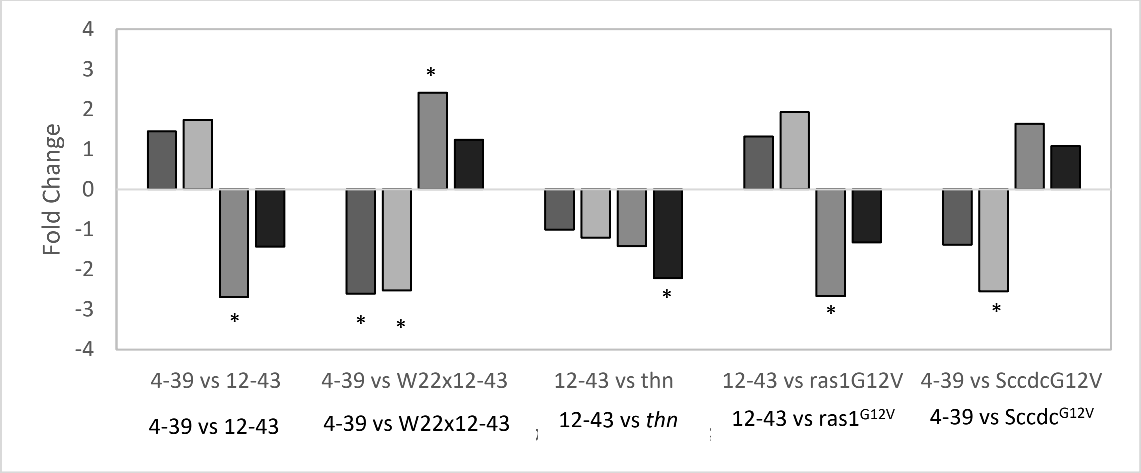

Figure S5: Expression analysis of *S. commune brl* genes in wild-type and mutant strains. (*) Significant differences ≥ 2-fold regulation.

Table S4: Sequence identity of *brl* genes in several *S. commune* strains compared to the respectively sequence of *S. commune* H4-8.

Gene	V153-21	4-39	4-40	E6	T2	T1
brl1 (530 bp)	99.5	99.6	90.3	90.2	100	n.a.
brl2	99.4	99.6	n.a.	n.a.	100	n.a.
brl3	98.7	99.6	n.a.	n.a.	100*	96.0*
brl4	99.7	99.4	100	100°	100	100

n.a. no amplicon; * 500 bp amplicon of *brl3*; ° 736 bp amplicon of *brl4*

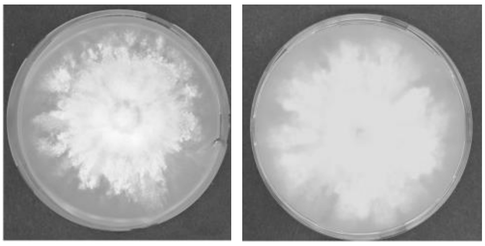

Figure S6: Morphology of dikaryotic *S. commune* strain 12-43x4-39.

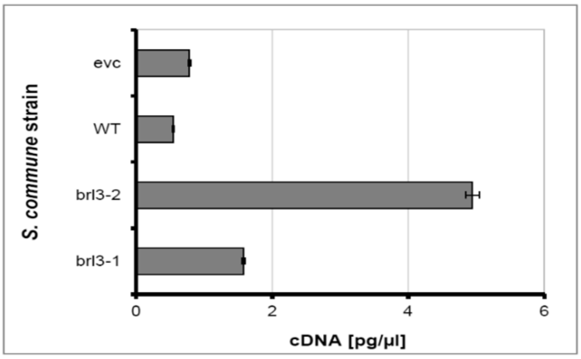

Figure S7: Absolute quantification by qRT-PCR of *brl3* expression. Overexpression of *brl3* was measured in strain brl3-1 (1.58 pg/µl), strain brl3-2 (4.94 pg/µl), WT (0.55 pg/µl) and evc (0.78 pg/µl).

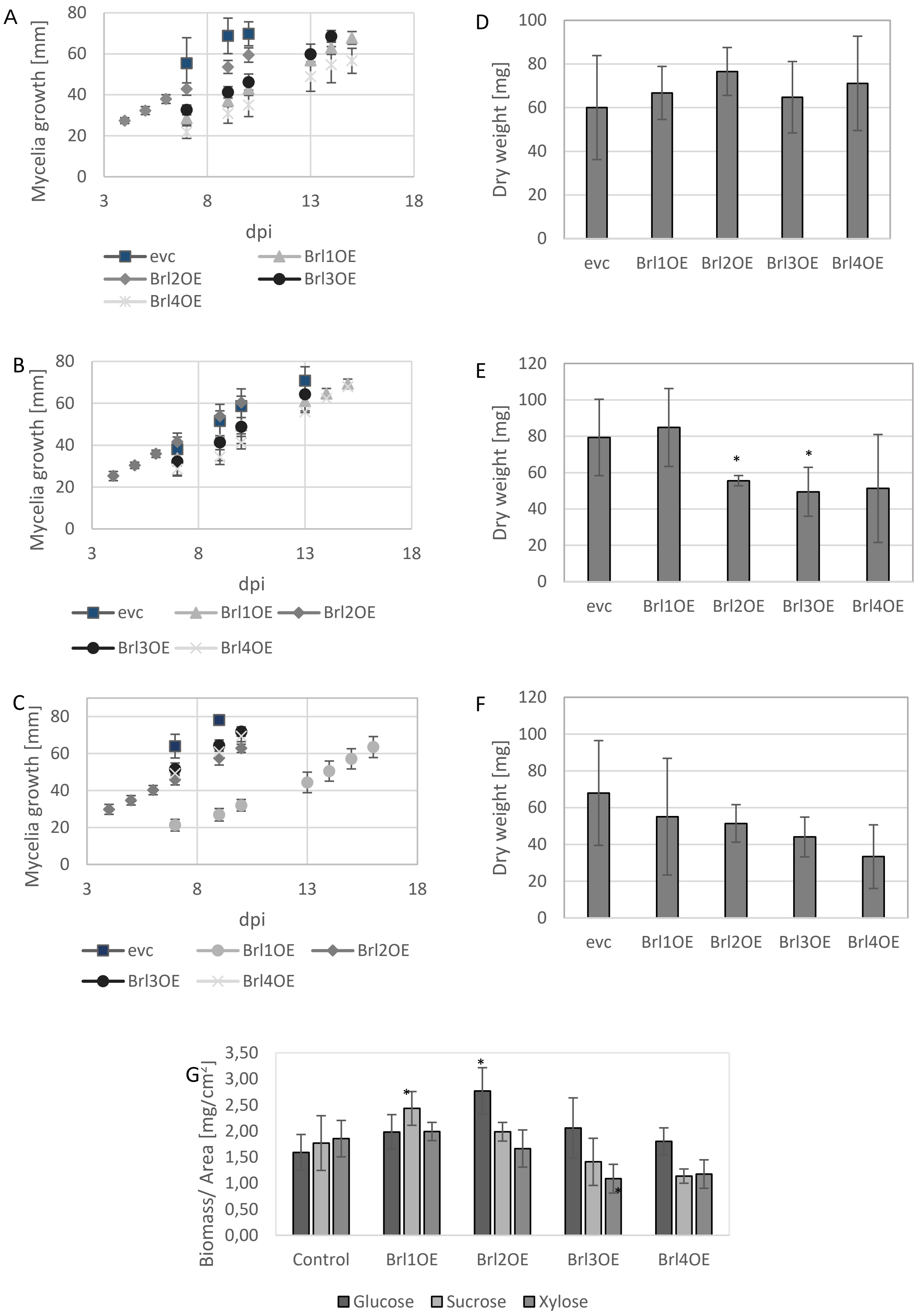

Figure S8: Growth (A-C) and biomass (D-F) of the empty vector control strain and *brl* overexpression strains using glucose (A, D), sucrose (B, E), and xylose (C, F) as carbon source. (G) shows the formed biomass/cm^2 on glucose, sucrose and xylose. (*) The student's T-test was used to determine the *P* value between control and transformants (*) $P < 0.05$.

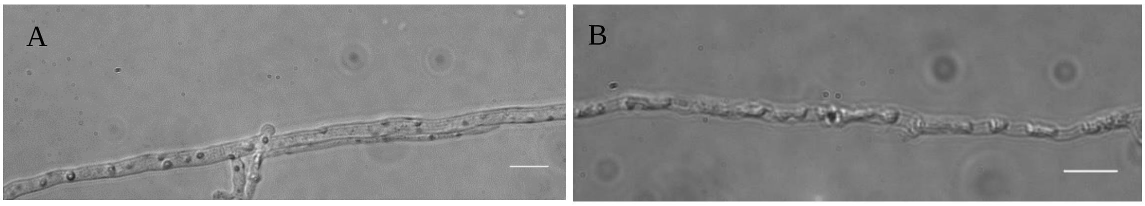

Figure S9: *S. commune* brl1OE (A) and brl4OE (B) form vacuole-rich wide hyphae. Bar represents 10 µm.

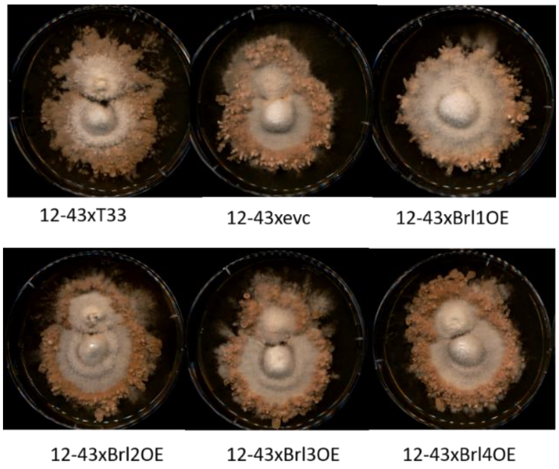

Figure S10: Mating interactions of T33, evc and *brl*-overexpressing strains with the compatible partner 12-43.

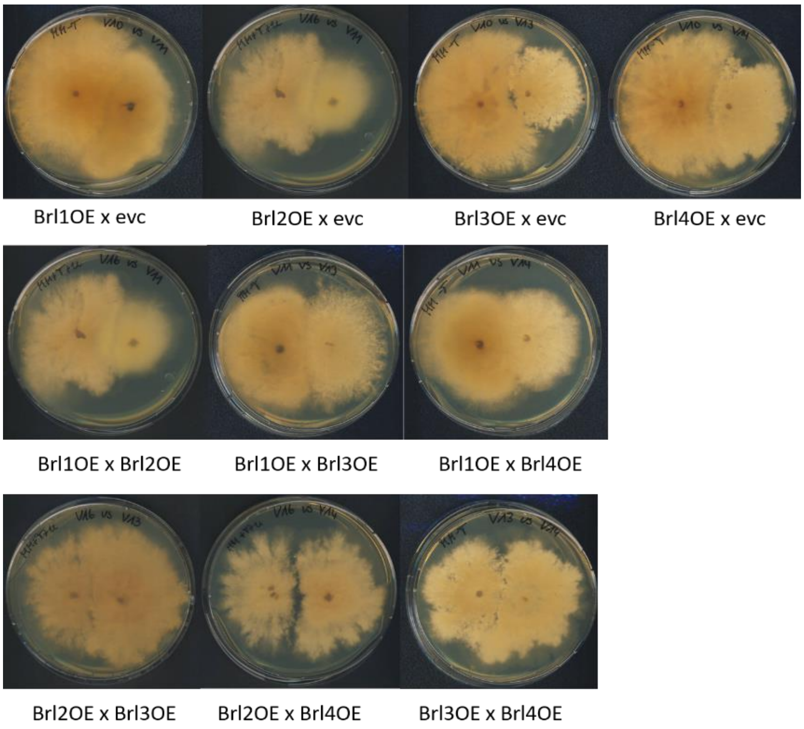

Figure S11: Confrontation of Brl1OE, Brl2OE, Brl3OE and Brl4OE with each other did not result in any significant growth reduction. Only confrontation of Brl2OE with Brl4OE resulted in a minor growth gap (not significant).

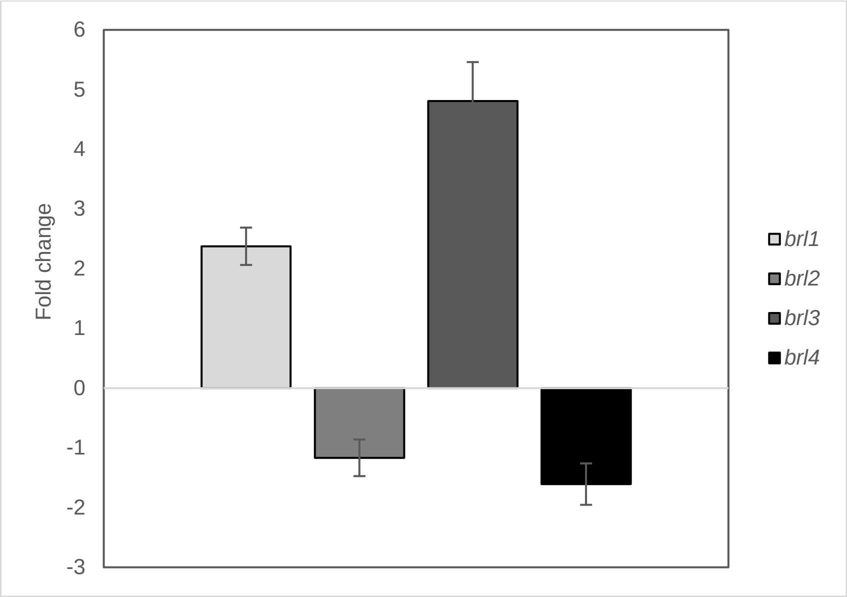

Figure S12: Expression of *brl1*, *brl2, brl3* and *brl4* in *S. commune* V153-21 (B_{null}). The data were normalized to the expression in the monokaryon 12-43 and relatively quantified to three reference genes.

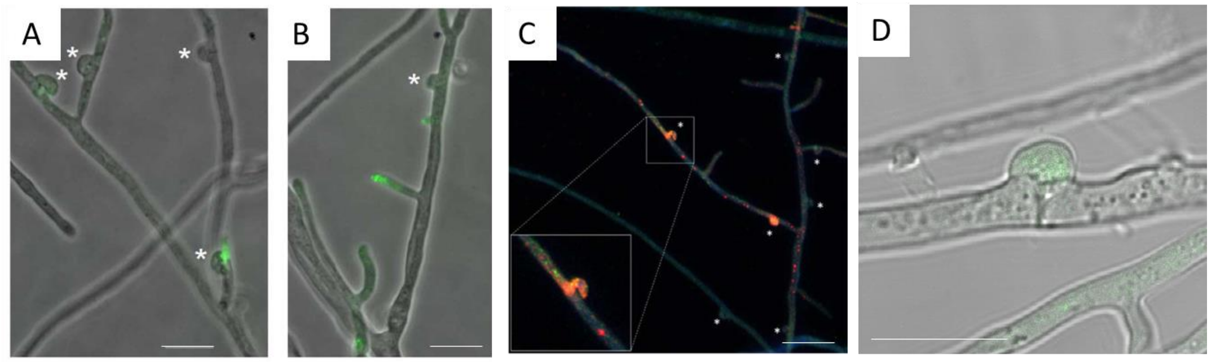

Figure S13. Localization of Brl2::his (A-C) and Bar2::egfp in dikaryon Localization of His-tagged Brl2 close to the hyphal tip and most likely membrane associated (A-B). Co-localization of Brl2::his and Bar2::HA is presented in dikaryotic hyphae (C). The labelling for Bar2 is shown in red, Brl2 in green and DAPI signal in blue. Localization of Bar2::egfp in dikaryon (D). Pseudoclamps are marked with asterisks. Bar represents 10 µm.
